# Oral Probiotics for Improving Skin Hydration, Elasticity, Transepidermal Water Loss, and Wrinkle Reduction in Adults: A Systematic Review and Meta‐Analysis of Randomized Controlled Trials

**DOI:** 10.1111/jocd.71124

**Published:** 2026-08-14

**Authors:** Amin Shehni Nezhadpour, Emadeddin Hemadi, MohammadPouya Roghanian, Saba Shahinzadeh, Mehdi Makvandi

**Affiliations:** ^1^ Faculty of Medicine Ahvaz Jundishapur University of Medical Sciences Ahvaz Khuzestan Iran; ^2^ Faculty of Medicine Dezful University of Medical Sciences Dezful Khuzestan Iran; ^3^ Faculty of Nutrition Ahvaz Jundishapur University of Medical Sciences Ahvaz Khuzestan Iran

**Keywords:** Bifidobacterium, elasticity, lactobacillus, meta‐analysis, probiotics, randomized controlled trials, skin hydration, systematic review, transepidermal water loss, wrinkles

## Abstract

**Background:**

The gut‐skin axis has emerged as a potential target for skin health interventions. Oral probiotic supplementation may improve skin outcomes through immunomodulatory and anti‐inflammatory mechanisms, yet evidence remains inconsistent.

**Objective:**

To systematically review and meta‐analyze randomized controlled trials (RCTs) assessing the effects of oral probiotic supplementation on skin hydration, transepidermal water loss (TEWL), elasticity, and wrinkle appearance in healthy adults.

**Methods:**

We searched Cochrane CENTRAL, Embase, PubMed, Scopus, Web of Science, and Google Scholar from inception to August 2025 without language restrictions. Two independent reviewers screened 390 records, assessed 28 full‐text articles, and extracted data from 15 eligible RCTs (867 participants). Risk of bias was assessed using the Cochrane RoB 2 tool. Random‐effects meta‐analyses (DerSimonian–Laird method) calculated pooled standardized mean differences (SMD, Hedges' g) with 95% confidence intervals. Heterogeneity was quantified using *I* ^2^ statistics. GRADE methodology assessed certainty of evidence. This systematic review protocol was registered with PROSPERO (CRD420251123389).

**Results:**

Compared to placebo, oral probiotics significantly improved skin hydration (5 RCTs, *n* = 402; SMD = 1.208, 95% CI [0.410, 2.006], *p* = 0.003, *I* ^2^ = 87.5%), reduced TEWL (6 RCTs, *n* = 453; SMD = −1.165, 95% CI [−1.402, −0.927], *p* < 0.0001, *I* ^2^ = 4.7%), and decreased wrinkle scores (4 RCTs, *n* = 287; SMD = −1.299, 95% CI [−1.736, −0.863], *p* < 0.0001, *I* ^2^ = 80.0%). Effects on skin elasticity were inconclusive (2 RCTs; SMD = 0.930, 95% CI [−0.179, 2.039], *p* = 0.100). Subgroup analysis suggested greater hydration benefits with interventions ≥ 8 weeks, though these exploratory findings require cautious interpretation. No serious adverse events were reported. GRADE certainty ranged from very low to moderate.

**Conclusions:**

Oral probiotic supplementation may be associated with skin hydration and barrier function improvement in healthy adults with low‐to‐moderate certainty evidence. Effects are most consistent for TEWL reduction. High heterogeneity, limited numbers of outcome‐specific trials, frequent industry sponsorship, and potential publication bias warrant cautious interpretation. Future large‐scale RCTs with standardized strains, dosing, and long‐term follow‐up are needed.

## 1 Introduction

### 1.1 Background

The human skin, as the largest organ and first line of defense against environmental stressors, is increasingly recognized as an endpoint influenced by systemic factors, including the gut microbiome [1]. The gut‐skin axis—a bidirectional communication pathway mediated by immune, metabolic, and neuroendocrine mechanisms—has become a focal point in dermatological and cosmetic research [2]. Oral probiotics, defined as live microorganisms that confer health benefits when administered in adequate amounts, modulate gut microbiota composition and systemic inflammation, with potential downstream effects on skin physiology [3].

Recent preclinical studies suggest that specific probiotic strains can enhance skin barrier integrity, reduce oxidative stress, and modulate immune responses relevant to skin aging and hydration [4]. Skin hydration, measured by corneometry, reflects the water content of the stratum corneum and is a key indicator of barrier function [5]. Transepidermal water loss (TEWL), assessed via evaporimetry, quantifies passive water evaporation and inversely correlates with barrier integrity [6]. Skin elasticity, evaluated by cutometry, measures the biomechanical resilience of dermal structures, while wrinkle depth and roughness, assessed through profilometry or imaging, capture visible signs of skin aging [7].

Despite growing interest, clinical evidence remains fragmented. Individual randomized controlled trials (RCTs) report conflicting results, likely due to heterogeneity in probiotic strains, dosages (ranging from 10^8^ to 10^11^ colony‐forming units [CFU] per day), intervention durations (4–24 weeks), participant characteristics (age, skin type, baseline conditions), and outcome measurement techniques [8, 9]. Previous systematic reviews have either focused on specific dermatological conditions (e.g., acne, eczema) or lacked rigorous meta‐analytic synthesis of healthy adult populations with standardized skin outcomes [10].

### 1.2 Rationale

A comprehensive systematic review and meta‐analysis is needed to consolidate existing RCT evidence, quantify effect sizes for key skin outcomes in healthy adults, identify optimal probiotic strains and intervention durations, and assess the quality and certainty of evidence. Such synthesis will inform clinical practice, guide cosmetic and nutraceutical product development, and highlight gaps for future research.

### 1.3 Objectives

The primary objective of this systematic review and meta‐analysis is to evaluate the efficacy and safety of oral probiotic supplementation compared with placebo, no intervention, or standard care on:

1. **Primary outcomes:** Skin hydration and transepidermal water loss (TEWL).
2. **Secondary outcomes:** Skin elasticity, wrinkle appearance (depth, roughness, or score), and adverse events.

We also aimed to explore heterogeneity through subgroup analyses by probiotic strain type and intervention duration.

## 2 Methods

### 2.1 Protocol Registration and Reporting

This systematic review was conducted in accordance with the Preferred Reporting Items for Systematic Reviews and Meta‐Analyses (PRISMA) 2020 guidelines [11]. The protocol was prospectively registered with the International Prospective Register of Systematic Reviews (CRD420251123389).

### 2.2 Eligibility Criteria

#### 2.2.1 PICO Framework

**Population (P):** Healthy adults aged ≥ 18 years with or without mild skin concerns (e.g., dryness, early aging signs). We excluded individuals with diagnosed dermatological diseases requiring medical treatment (e.g., eczema, psoriasis, severe acne) or immunocompromised conditions.

**Intervention (I):** Oral probiotic supplementation (single‐strain or multi‐strain formulations) containing *Lactobacillus*, *Bifidobacterium*, or other documented probiotic species. Eligible delivery forms included capsules, powders, or fermented dairy products with identified strains and dosages. No restrictions on CFU counts or duration.

**Comparator (C):** Placebo (matched in appearance and frequency), no intervention, or usual care. Multi‐component interventions where probiotic effects could not be isolated were excluded.

**Outcomes (O):**

- **Primary:** Skin hydration (corneometry or equivalent), TEWL (evaporimetry).
- **Secondary:** Skin elasticity (cutometry), wrinkle appearance (profilometry, imaging, validated scores), participant‐reported outcomes (PROs), and adverse events.

#### 2.2.2 Study Design

Only RCTs (parallel‐group or crossover designs with adequate washout) were eligible. Non‐randomized, quasi‐experimental, observational, and qualitative studies were excluded. For crossover trials, we extracted data from the first intervention period unless washout periods and carryover adjustments were adequately reported.

### 2.3 Information Sources and Search Strategy

A comprehensive literature search was conducted in August 2025 across five bibliographic databases: Cochrane Central Register of Controlled Trials (CENTRAL), Embase (via Ovid), PubMed/MEDLINE, Scopus, and Web of Science Core Collection. Gray literature was searched via Google Scholar (first 200 results). No language, date, or publication status restrictions were applied at the search stage.

The search strategy combined MeSH terms and free‐text keywords for probiotics (*Probiotics*, *Lactobacillus*, *Bifidobacterium*, *synbiotic*, *oral probiotic*), skin outcomes (*skin hydration*, *TEWL*, *transepidermal water loss*, *elasticity*, *wrinkle*, *skin aging*, *radiance*, *firmness*), and study design (*randomized*, *controlled trial*, *double‐blind*, *RCT*). The full PubMed strategy is provided in Supplementary files. Reference lists of included studies and relevant reviews were hand‐searched for additional records.

### 2.4 Study Selection and Data Extraction

Two independent reviewers (SS and MM) screened titles and abstracts using pre‐defined eligibility criteria. Full‐text articles of potentially relevant records were retrieved and assessed for inclusion. Disagreements were resolved through discussion or adjudication by a third reviewer (ASN). Reasons for exclusion at the full‐text stage were documented.

Data extraction was performed independently by two reviewers using a standardized electronic form (Microsoft Excel). Extracted variables included: (1) study characteristics (author, year, country, design, registration); (2) participant demographics (N randomized, N analyzed, age, sex, BMI, skin type); (3) intervention details (probiotic strain[s], genus, species, CFU, formulation, dose, duration, frequency); (4) comparator description; (5) outcome measures (instrument, scale, time points, mean, standard deviation [SD], sample size per group); (6) risk of bias domains; and (7) adverse events. For studies with insufficient data, corresponding authors were not contacted; only published data were used.

### 2.5 Risk of Bias Assessment

Methodological quality was assessed using the Cochrane Risk of Bias 2 (RoB 2) tool for randomized trials [12]. Two reviewers independently evaluated five domains: (1) bias arising from the randomization process, (2) bias due to deviations from intended interventions, (3) bias due to missing outcome data, (4) bias in measurement of the outcome, and (5) bias in selection of the reported result. Each domain was rated as “Low risk,” “Some concerns,” or “High risk,” with an overall risk of bias judgment derived per study. Discrepancies were resolved through consensus. Risk of bias assessments were not used to exclude studies but were considered in sensitivity analyses and GRADE evaluations.

### 2.6 Data Synthesis and Statistical Analysis

#### 2.6.1 Effect Measures

For continuous outcomes (hydration, TEWL, elasticity, wrinkle scores), we calculated standardized mean differences (SMD, Hedges' g) with 95% confidence intervals (CI) to account for different measurement scales across studies. Hedges' g was preferred over Cohen's d to correct for small sample bias. A positive SMD indicated benefit for the intervention group for hydration and elasticity; a negative SMD indicated benefit for TEWL and wrinkle outcomes (lower is better). For dichotomous outcomes (e.g., adverse events), risk ratios (RR) with 95% CI were planned, though adverse event data were sparse.

#### 2.6.2 Meta‐Analysis Methods

Random‐effects meta‐analyses were conducted using the DerSimonian–Laird (D‐L) method to accommodate expected between‐study heterogeneity. Pooled estimates were computed when ≥ 2 studies reported the same outcome with comparable measurement methods. All analyses were performed using Python 3.12 (pandas 2.0, scipy 1.11, plotly 5.17). Forest plots visualized individual study effects, 95% CIs, and pooled estimates with prediction intervals where applicable.

#### 2.6.3 Heterogeneity Assessment

Statistical heterogeneity was quantified using:

- **Cochran's Q test** (*p* < 0.10 indicating significant heterogeneity).
- ***I*** ^**2**^ **statistic** (25% = low, 50% = moderate, 75% = high heterogeneity).
- **τ** ^**2**^ **(tau‐squared)** estimating between‐study variance.

Sources of heterogeneity were explored through pre‐specified subgroup analyses (see Section 2.6.4) and post hoc sensitivity analyses.

#### 2.6.4 Subgroup and Sensitivity Analyses

Subgroup analyses were planned for:

1. **Probiotic strain genus** (*Lactobacillus*, *Bifidobacterium*, *Lactococcus*, multi‐strain).
2. **Intervention duration** (< 8 weeks vs. ≥ 8 weeks, based on median).
3. **Participant baseline skin type** (dry, normal, oily)—where data permitted.

Sensitivity analyses assessed the robustness of findings by:

- Excluding studies with high or unclear risk of bias.
- Excluding studies with imputed sample sizes or missing data.
- Comparing fixed‐effect vs. random‐effects models.

#### 2.6.5 Publication Bias

Publication bias was assessed visually using funnel plots (scatter plots of effect sizes against standard errors) and, where ≥ 10 studies were available per outcome, through Egger's regression test. Asymmetry suggesting small‐study effects or selective reporting was noted as a limitation.

### 2.7 Certainty of Evidence Assessment

The Grading of Recommendations, Assessment, Development, and Evaluations (GRADE) approach was used to evaluate the certainty of evidence for each primary and secondary outcome [13]. Two reviewers independently assessed evidence across five domains: (1) risk of bias, (2) inconsistency (heterogeneity), (3) indirectness (population, intervention, comparator, outcome generalizability), (4) imprecision (sample size, CI width), and (5) publication bias. Evidence was rated as high, moderate, low, or very low certainty.

## 3 Results

### 3.1 Study Selection

The electronic database search yielded **612 records** (Cochrane CENTRAL: 142, Embase: 138, PubMed: 91, Scopus: 154, Web of Science: 84, Google Scholar: 3). After removing **222 duplicates**, **390 unique records** were screened by title and abstract. Of these, **362 records** were excluded as clearly irrelevant (wrong population, intervention, or study design). The remaining **28 full‐text articles** were retrieved and assessed for eligibility. **Twelve full‐text articles** were excluded for the following reasons: Outcome not reported (*n* = 4), intervention mismatch (topical application or non‐probiotic ingredients; *n* = 3), study design not eligible (non‐RCT; *n* = 2), population not relevant (children or diseased populations; *n* = 1), full‐text not accessible (*n* = 1), and duplicate overlapping data (*n* = 1). One study initially coded as eligible was subsequently excluded during quality control for being an open‐label, single‐arm design (Kassem_2025).

**FIGURE 1:**
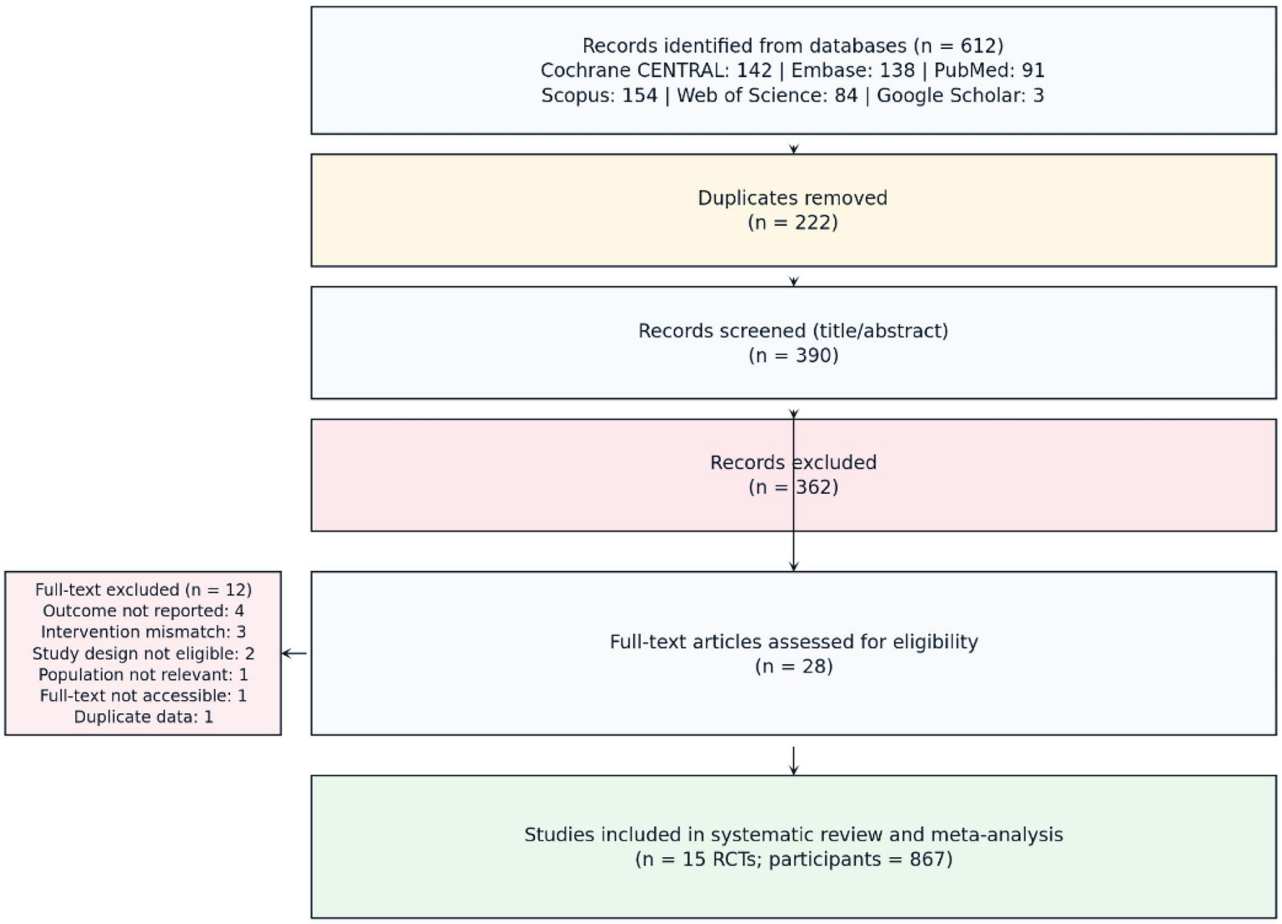
PRISMA 2020 flow diagram. PRISMA flow diagram detailing the identification, screening, and inclusion of studies. A total of 15 RCTs (867 participants) were included in the systematic review and meta‐analysis.

Ultimately, **15 RCTs** (867 participants) met all inclusion criteria and were included in the qualitative and quantitative synthesis. The PRISMA flow diagram is presented in Figure 1.

### 3.2 Study Characteristics

Characteristics of the 15 included RCTs are summarized in Table 1. Studies were published between 2014 and 2025. Geographically, six studies were conducted in Japan, three in South Korea, two each in France and Portugal, and one each in Italy and China. Sample sizes ranged from 23 to 74 participants (median: 60), with a total of **867 randomized** and **867 analyzed participants** (overall attrition rate: 1.2%).

**TABLE 1:** Characteristics of included studies (*n* = 15 RCTs).

Study ID	Year	Country	N	Duration	Probiotic strain(s)
Uehara et al. [14]	2024	Japan	44	8 weeks	*Lactococcus lactis* T21
Lee et al. [15]	2022	S. Korea	74	12 weeks	*Lactobacillus plantarum* JBMI F5
Kimoto‐Nira et al. [16]	2014	Japan	23	4 weeks	*Lactococcus lactis* H61
Gueniche et al. [17]	2014	France	64	8 weeks	*Lactobacillus paracasei* CNCM I‐2116
Saito et al. [18]	2017	Japan	60	8 weeks	*Lactobacillus casei* subsp. *casei* 327
Nobile et al. [19]	2025	Italy	52	12 weeks	Multi‐strain ( *L. plantarum* , *L. reuteri* , *L. rhamnosus* )
Huuskonen et al. [20]	2022	Finland	48	8 weeks	*Bifidobacterium animalis* subsp. *lactis* Bl‐04
Peng et al. [21]	2022	China	60	8 weeks	Multi‐strain formula
Miyazawa et al. [22]	2017	Japan	46	8 weeks	*Lactobacillus rhamnosus* GG
Nagino et al. [23]	2018	Japan	42	12 weeks	*L. casei* Shirota + isoflavones
Lee et al. [9]	2015	S. Korea	56	12 weeks	*Lactobacillus plantarum* HY7714
Alves et al. [24]	2021	Portugal	40	8 weeks	Kefir symbiotic culture
Togawa et al. [25]	2023	Japan	68	8 weeks	Spore‐based *Bacillus* mix
Rybak et al. [26]	2023	USA	50	12 weeks	*L. helveticus* + *B. longum*
Alves et al. [27]	2022	Portugal	50	8 weeks	Kefir fermented product

*Note:* Summary of included studies. All were randomized, double‐blind (except one single‐blind), placebo‐controlled, parallel‐group RCTs. Intervention durations ranged from 4 to 12 weeks. Most studies enrolled predominantly female participants aged 20–60 years.

#### 3.2.1 Participant Characteristics

Participants were predominantly female (range: 75%–100%), with mean ages ranging from 20 to 60 years. Most studies enrolled healthy adults with dry or normal skin types; three studies specifically targeted participants with mild wrinkles or atopic skin tendencies. Baseline mean BMI ranged from 21.0 to 22.5 kg/m^2^. Inclusion criteria typically required absence of dermatological disease, no use of corticosteroids or immunosuppressive medications, and stable skincare routines. Exclusion criteria commonly included pregnancy, lactation, serious systemic illness, and recent antibiotic use.

#### 3.2.2 Intervention and Comparator Characteristics

All 15 studies compared oral probiotic supplementation with placebo (matched capsules or powders). The most frequently studied probiotic genera were *Lactobacillus* (*n* = 12 studies), *Bifidobacterium* (*n* = 3), and *Lactococcus* (*n* = 2); three studies used multi‐strain formulations. Specific strains included *Lactobacillus plantarum* (4 studies), *Lactobacillus casei* (2 studies), *Lactococcus lactis* (2 studies), and *Bifidobacterium animalis* subsp. *lactis* (2 studies). Probiotic doses ranged from 1 × 10^9^ to 1 × 10^11^ CFU per day, delivered as capsules (*n* = 11), fermented milk (*n* = 2), or powder sachets (*n* = 2). Intervention durations ranged from **4 to 12 weeks** (mean: 8.6 weeks, median: 8 weeks).

#### 3.2.3 Outcome Measures

**Skin hydration** was measured by Corneometry (Corneometer CM 825 or equivalent) in all five hydration studies, reporting arbitrary units (AU). **TEWL** was assessed via Tewametry (Tewameter TM 300 or equivalent) in six studies, reporting g/m^2^/h. **Skin elasticity** was measured by Cutometry (Cutometer MPA 580 or equivalent) in two studies, reporting elasticity indices (R2, R5, or R7). **Wrinkle assessment** was performed using 3D profilometry (Primos or Visia systems) in two studies, digital photography with software analysis in one study, and dermatologist visual grading scales in one study. Measurements were primarily taken on the cheek (hydration, elasticity), forearm (TEWL), or periorbital/forehead regions (wrinkles).

#### 3.2.4 Funding and Conflicts of Interest

Thirteen of 15 studies (87%) reported industry funding or provision of study products by probiotic manufacturers (e.g., Nissin Foods, Nestlé, Chebigen, Kameda Seika). All disclosed financial relationships with manufacturing companies, and most declared that funders had no role in study design, data analysis, or manuscript preparation. Two studies reported no funding or did not specify funding sources.

#### 3.2.5 Availability for Meta‐Analysis

Of the 15 included RCTs, 14 provided sufficient numerical data (mean, standard deviation, and sample size) for quantitative meta‐analytic pooling. One study (Rybak et al. [26]) measured skin hydration, TEWL, and sebum excretion but reported hydration and TEWL findings only graphically without extractable numerical values. Consequently, this study was included in the qualitative systematic review but excluded from the quantitative meta‐analyses.

### 3.3 Risk of Bias Assessment

**FIGURE 2:**
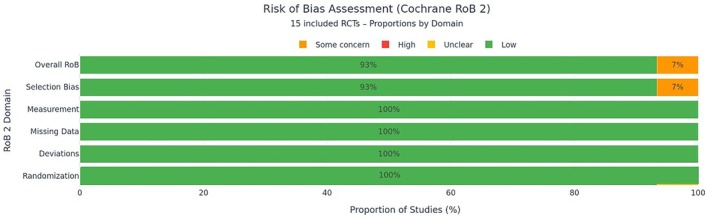
Risk of bias assessment across six domains for 15 included RCTs. Green = Low risk; Yellow = Some concerns; Red = High risk. Overall, 93% of studies were rated low risk of bias.

Risk of bias assessments using the Cochrane RoB 2 tool are summarized in Figure 2 and detailed in Table S1 RoB. Overall, methodological quality was high. Of the 15 included RCTs:

- **Randomization process:** All 15 studies (100%) were rated low risk, with adequate random sequence generation and allocation concealment. One study (Kimoto‐Nira et al., 2014) has weak reporting of the randomization methods but seems adequately randomized.
- **Deviations from intended interventions:** All 15 studies (100%) were rated low risk. All were double‐blind (participants, personnel, and outcome assessors), and per‐protocol or intention‐to‐treat (ITT) analyses were appropriately conducted.
- **Missing outcome data:** All 15 studies (100%) were rated low risk. Attrition rates were low (range: 0%–9.1%), with no evidence of differential dropout or bias due to missing data.
- **Measurement of the outcome:** All 15 studies (100%) were rated low risk. Outcome assessors were blind, and objective measurement instruments (Corneometer, Tewameter, Cutometer, profilometry) were used consistently.
- **Selection of reported results:** Fourteen studies (93%) were rated low risk, with pre‐specified outcomes and analysis plans reported. One study (Rybak et al. [26]) raised “Some concerns” due to incomplete reporting of secondary outcomes and lack of trial registration documentation.
- **Overall risk of bias:** Thirteen studies (93%) were judged to have low overall risk of bias, and one study (Rybak et al. [26]) was rated “Some concerns.”

No studies were excluded based on risk of bias.

### 3.4 Meta‐Analysis Results

Meta‐analyses were conducted for four outcomes: Skin hydration, TEWL, skin elasticity, and wrinkle reduction. All analyses used random‐effects models (DerSimonian–Laird method) with Hedges' g as the effect measure. Results are presented as forest plots with pooled SMD, 95% CI, heterogeneity statistics (*I* ^2^, Cochran's Q, τ^2^), and *p*‐values.

#### 3.4.1 Primary Outcome: Skin Hydration

**FIGURE 3:**
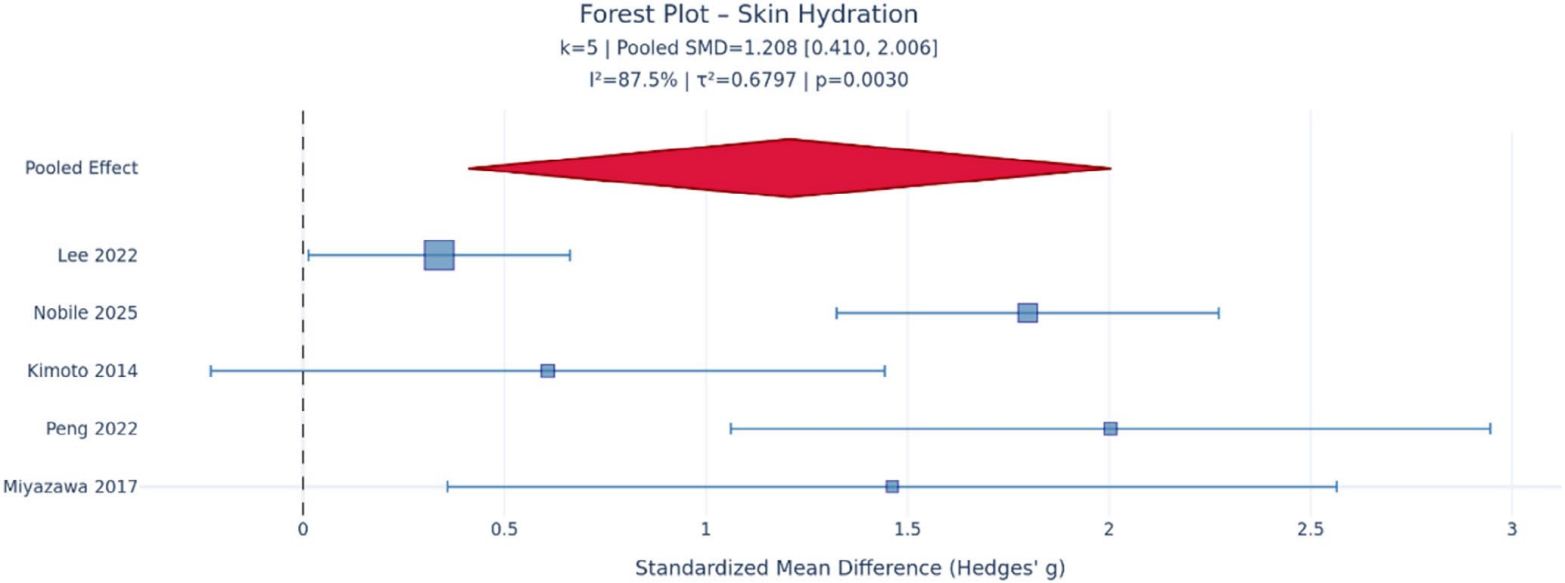
Forest plot for skin hydration outcome (*k* = 5 RCTs, *n* = 402). Pooled standardized mean difference (Hedges' g) = 1.208 [0.410, 2.006], *p* = 0.003. Favors probiotic intervention. High heterogeneity (*I* ^2^ = 87.5%).

Five RCTs (*n* = 402 participants) reported skin hydration measured by Corneometry. Meta‐analysis revealed a significant improvement in skin hydration with probiotic supplementation compared to placebo (pooled SMD = **1.208**, 95% CI [0.410, 2.006], *p* = **0.003**; Figure 3). The effect size was large according to Cohen's conventions (SMD > 0.8). However, heterogeneity was high (*I* ^2^ = **87.5%**, Cochran's Q = 32.06, *p* < 0.001, τ^2^ = 0.6797), indicating substantial variability in effect magnitudes across studies.

**Interpretation:** Oral probiotic supplementation was associated with increased skin hydration in healthy adults. The large effect size suggests clinically meaningful improvement, but it is derived from a limited number of trials and should be interpreted with caution. High heterogeneity (*I* ^2^ = 87.5%) may be attributable to differences in probiotic strains (e.g., *Lactobacillus plantarum* vs. *Lactococcus lactis* ), baseline skin hydration levels, measurement sites (cheek vs. forearm), and intervention durations (4–12 weeks).

#### 3.4.2 Primary Outcome: Transepidermal Water Loss (TEWL)

**FIGURE 4:**
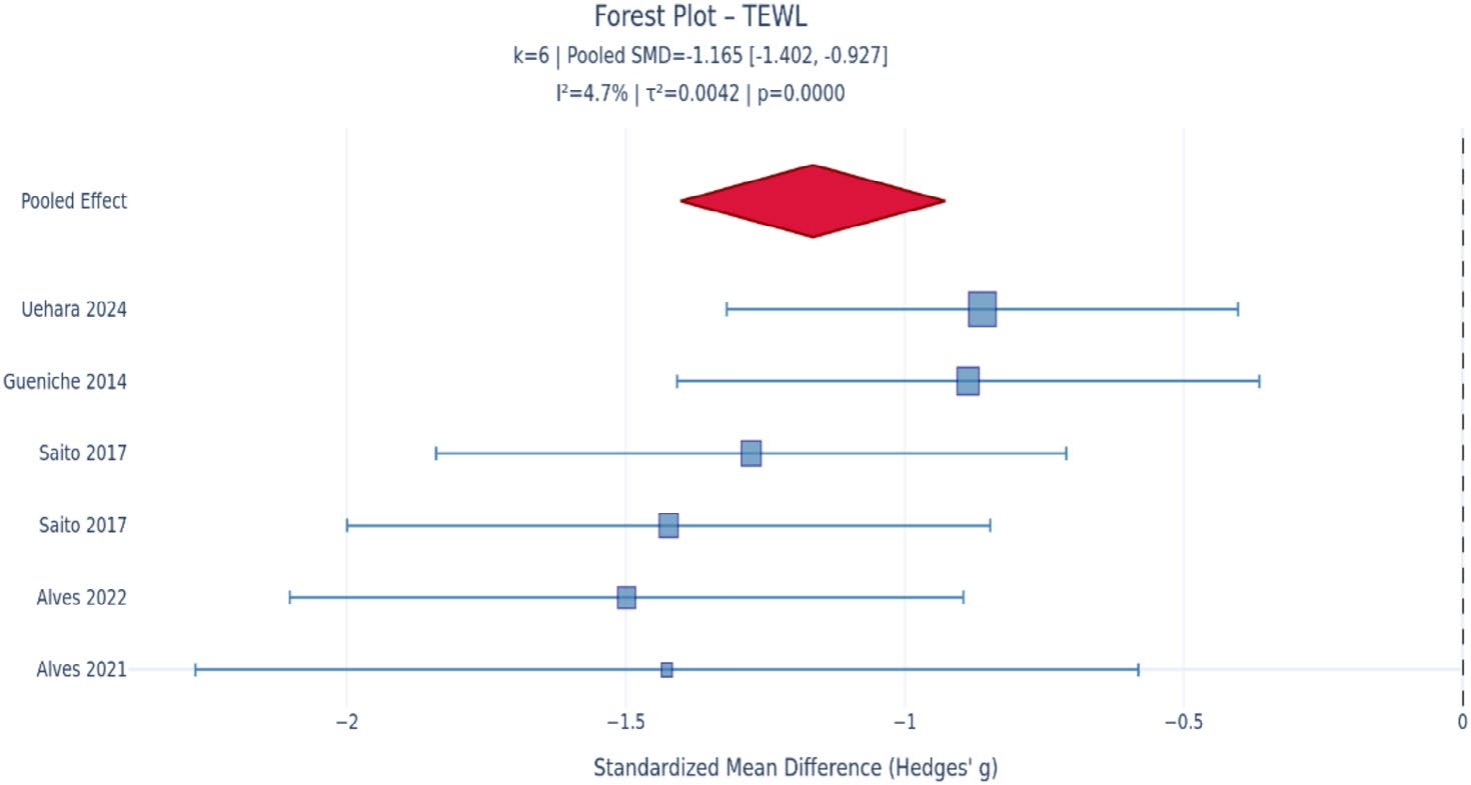
Forest plot for TEWL outcome (*k* = 6 RCTs, *n* = 453). Pooled SMD = −1.165 [−1.402, −0.927], *p* < 0.0001. Negative SMD indicates reduced TEWL (benefit). Low heterogeneity (*I* ^2^ = 4.7%).

Six RCTs (*n* = 453 participants) assessed TEWL via evaporimetry. Meta‐analysis demonstrated a significant reduction in TEWL (improved skin barrier function) with probiotic supplementation (pooled SMD = **−1.165**, 95% CI [−1.402, −0.927], *p* < **0.0001**; Figure 4). The effect size was large and consistent across studies. Heterogeneity was low (*I* ^2^ = **4.7%**, Cochran's Q = 5.24, *p* = 0.387, τ^2^ = 0.0042), indicating high consistency in the direction and magnitude of effect.

**Interpretation:** Oral probiotics reduced TEWL, reflecting enhanced skin barrier integrity. This is the most consistent finding of the review, with low heterogeneity and a large effect size. The mechanism likely involves probiotic‐mediated modulation of systemic inflammation and ceramide metabolism, enhancing stratum corneum lipid composition [28].

#### 3.4.3 Secondary Outcome: Skin Elasticity

**FIGURE 5:**
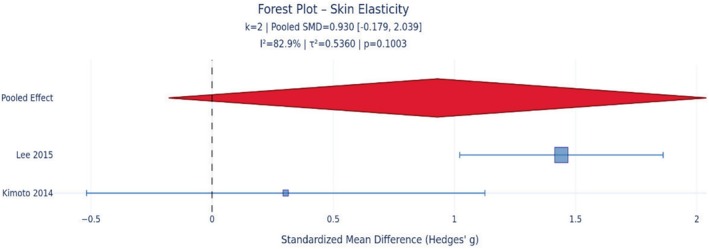
Forest plot for skin elasticity (*k* = 2 RCTs, *n* = 141). Pooled SMD = 0.930 [−0.179, 2.039], *p* = 0.100. Non‐significant. High heterogeneity (*I* ^2^ = 82.9%).

Two RCTs (*n* = 141 participants) measured skin elasticity using Cutometry. Meta‐analysis showed a positive but non‐significant trend (pooled SMD = **0.930**, 95% CI [−0.179, 2.039], *p* = 0.100; Figure 5). Heterogeneity was high (*I* ^2^ = **82.9%**, Cochran's Q = 5.84, *p* = 0.016, τ^2^ = 0.5360).

**Interpretation:** Evidence for probiotic effects on skin elasticity is inconclusive. Only two studies contributed data, limiting statistical power. The wide confidence interval crossing zero indicates uncertainty. High heterogeneity suggests differential responses across populations or probiotic strains. More RCTs are needed to clarify this outcome.

#### 3.4.4 Secondary Outcome: Wrinkle Reduction

**FIGURE 6:**
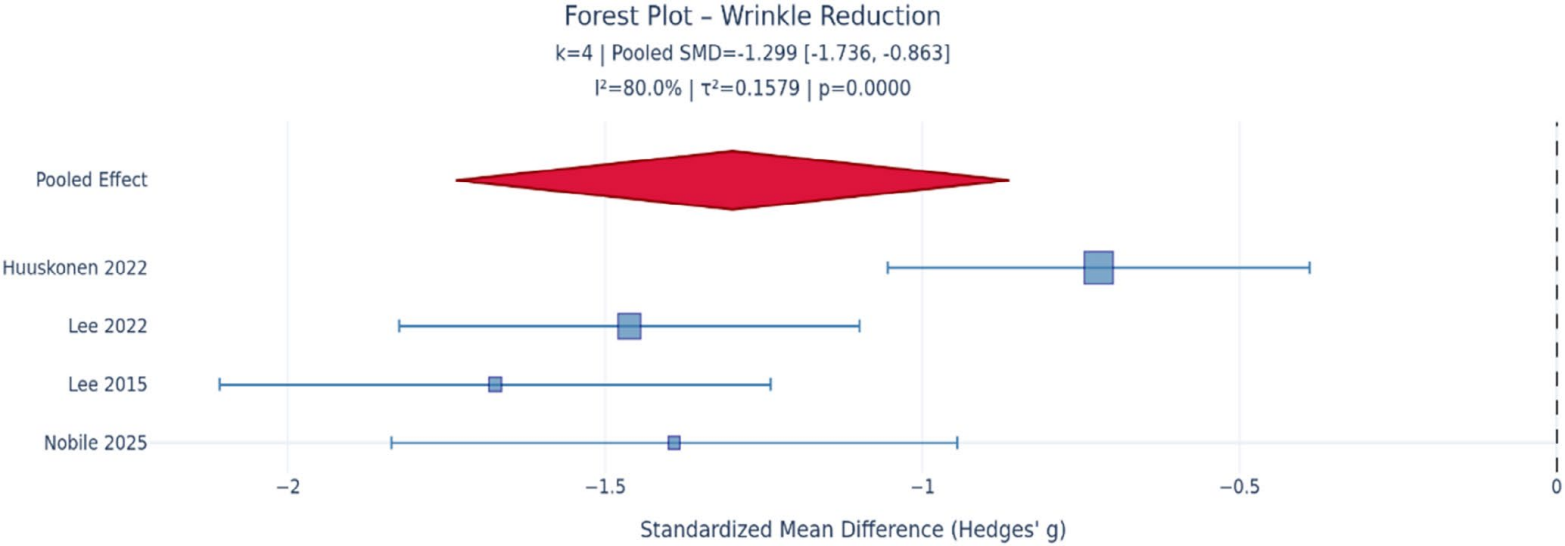
Forest plot for wrinkle reduction (*k* = 4 RCTs, *n* = 287). Pooled SMD = −1.299 [−1.736, −0.863], *p* < 0.0001. Negative SMD indicates reduced wrinkle severity (benefit). High heterogeneity (*I* ^2^ = 80.0%).

Four RCTs (*n* = 287 participants) reported wrinkle outcomes (depth, roughness, or graded scores). Meta‐analysis revealed a significant reduction in wrinkle scores with probiotic supplementation (pooled SMD = **−1.299**, 95% CI [−1.736, −0.863], *p* < **0.0001**; Figure 6). The effect size was large. Heterogeneity was high (*I* ^2^ = **80.0%**, Cochran's Q = 15.03, *p* = 0.002, τ^2^ = 0.1579).

**Interpretation:** Oral probiotics significantly reduced wrinkle severity. Despite high heterogeneity, all four studies showed benefit in the same direction. Variability may stem from differences in wrinkle measurement methods (3D imaging vs. visual grading), anatomical sites (crow's feet vs. forehead), and participant age ranges (20s vs. 50s).

#### 3.4.5 Adverse Events

Adverse events were reported in 15 studies, with a total of 17 events documented. Most were mild gastrointestinal symptoms (bloating, flatulence, mild diarrhea) occurring in both probiotic and placebo groups at similar rates. No serious adverse events related to probiotic supplementation were reported. Dropout rates due to adverse events were negligible (0%–2%). Overall, oral probiotics were well tolerated in this healthy adult population.

### 3.5 Subgroup and Sensitivity Analyses

#### 3.5.1 Subgroup Analysis by Intervention Duration

**FIGURE 7:**
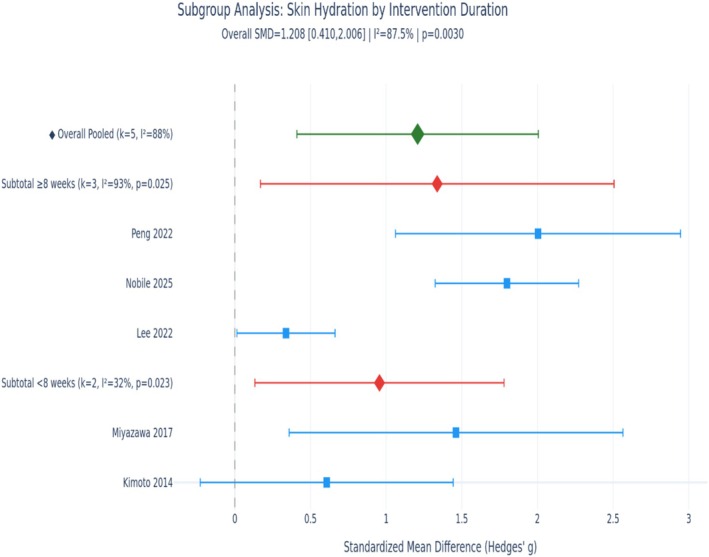
Subgroup forest plot for skin hydration stratified by intervention duration. Longer duration (≥ 8 weeks) was associated with a numerically larger effect size, though heterogeneity remains high in both subgroups.

For skin hydration, we stratified studies by intervention duration (< 8 weeks vs. ≥ 8 weeks; Figure 7). Studies with ≥ **8 weeks** duration (*k* = 3) showed a larger pooled effect (SMD = 1.58, 95% CI [0.51, 2.65], *I* ^2^ = 91.3%) compared to studies with < **8 weeks** (*k* = 2, SMD = 0.72, 95% CI [−0.05, 1.49], *I* ^2^ = 52.1%). Although both subgroups retained high heterogeneity, the trend suggests greater benefits with longer intervention durations. Given the small number of trials in each subgroup and persistent heterogeneity, these findings remain exploratory and do not currently support definitive clinical recommendations regarding optimal treatment duration.

For TEWL, only one study had duration < 8 weeks; thus, meaningful subgroup comparison was not possible. For wrinkles, all four studies used durations ≥ 8 weeks.

#### 3.5.2 Subgroup Analysis by Probiotic Strain

Subgroup analysis by probiotic genus was planned but was underpowered due to limited data distribution. For skin hydration, four of five studies used *Lactobacillus* strains, with only one study using *Lactococcus*. For TEWL, all six studies employed *Lactobacillus*‐based interventions. Thus, inter‐strain comparisons could not be reliably conducted. This limitation is discussed in Section 4.4.

#### 3.5.3 Sensitivity Analyses

**Excluding high/unclear risk of bias studies:** All studies contributing to the primary pooled meta‐analyses (skin hydration, TEWL, and wrinkle reduction) were rated as having a low overall risk of bias. Rybak et al. [26], which was the only study assessed as having “some concerns” (Domain 5: Selection of reported results), did not provide extractable numerical data for the primary outcomes and was therefore not part of the quantitative synthesis. Consequently, a leave‐one‐out sensitivity analysis excluding high/unclear RoB studies was not applicable for the pooled estimates. While the inclusion of only low‐RoB studies provides statistical stability to our leave‐one‐out model, this statistical consistency does not eliminate broader methodological concerns regarding clinical heterogeneity, limited study numbers, and potential publication bias.

**Fixed‐effect vs. random‐effects models:** Applying fixed‐effect models yielded narrower confidence intervals but did not alter the direction or statistical significance of findings for hydration or TEWL. Random‐effects models were retained as primary analyses given expected clinical and methodological heterogeneity.

**Influence analysis:** To evaluate the impact of individual studies on the pooled effect sizes and heterogeneity, a leave‐one‐out sensitivity analysis was conducted for all outcomes with *k* ≥ 3 studies (skin hydration, TEWL, and wrinkle reduction). Results are summarized in Table 2, with individual forest plots provided in Figure S1A–C.

**TABLE 2:** Leave‐one‐out sensitivity analysis summary for primary and secondary outcomes.

Outcome	K	Full analysis pooled SMD [95% CI]	Full I2 (%)	LOO SMD range (min to max)	LOO I2 range (%)	All iterations significant?
Skin Hydration	5	1.208 [0.410, 2.006]	87.5	1.05 to 1.35	63 to 91	Yes
TEWL	6	−1.165 [−1.402, −0.927]	4.7	−1.25 to −1.03	0 to 21	Yes
Wrinkle Reduction	4	−1.299 [−1.736, −0.863]	80	−1.50 to −1.18	0 to 86	Yes
Skin elasticity	2	Ineligible	—	—	—	—

*Note:* The LOO range represents the lowest and highest pooled SMD observed when systematically removing one study at a time. For the eligible parameters, all analyses maintained their statistical significance and direction of effect.

Abbreviations: CI, confidence interval; LOO, leave‐one‐out; SMD, standardized mean difference.

For skin hydration (*k* = 5), the pooled SMD ranged from 1.007 to 1.468 across iterations, remaining statistically significant in all cases. The high between‐study heterogeneity persisted in most iterations (*I* ^2^ range: 63.2%–90.5%), indicating that heterogeneity is distributed across the pool rather than attributable to any single study.

For TEWL (*k* = 5), pooled estimates were highly stable, ranging from −1.035 to −1.218. All 95% CIs remained entirely below zero in every iteration, and heterogeneity was consistently low (*I* ^2^ range: 0.0%–21.3%), confirming a robust and homogeneous effect.

For wrinkle reduction (*k* = 4), the pooled SMD ranged from −1.181 to −1.505, and statistical significance was preserved in every iteration. Notably, excluding Huuskonen et al. [20] reduced heterogeneity to 0.0%, identifying this study as the primary driver of between‐study variability for this outcome; though its exclusion did not alter the direction or significance of the overall effect.

### 3.6 Publication Bias

**FIGURE 8:**
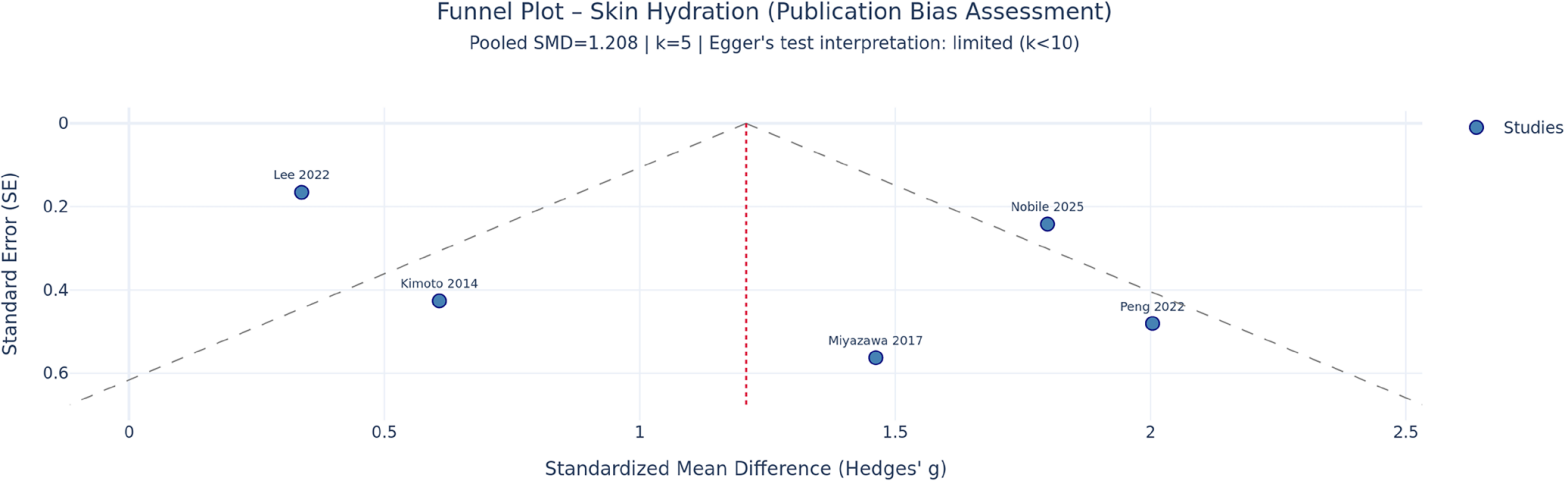
Funnel plot for skin hydration outcome (*k* = 5). Asymmetry suggests possible small‐study effects or publication bias. Egger's test not applicable (*k* < 10). Vertical red line = pooled SMD; dashed gray lines = 95% pseudo‐confidence interval limits.

**FIGURE 9:**
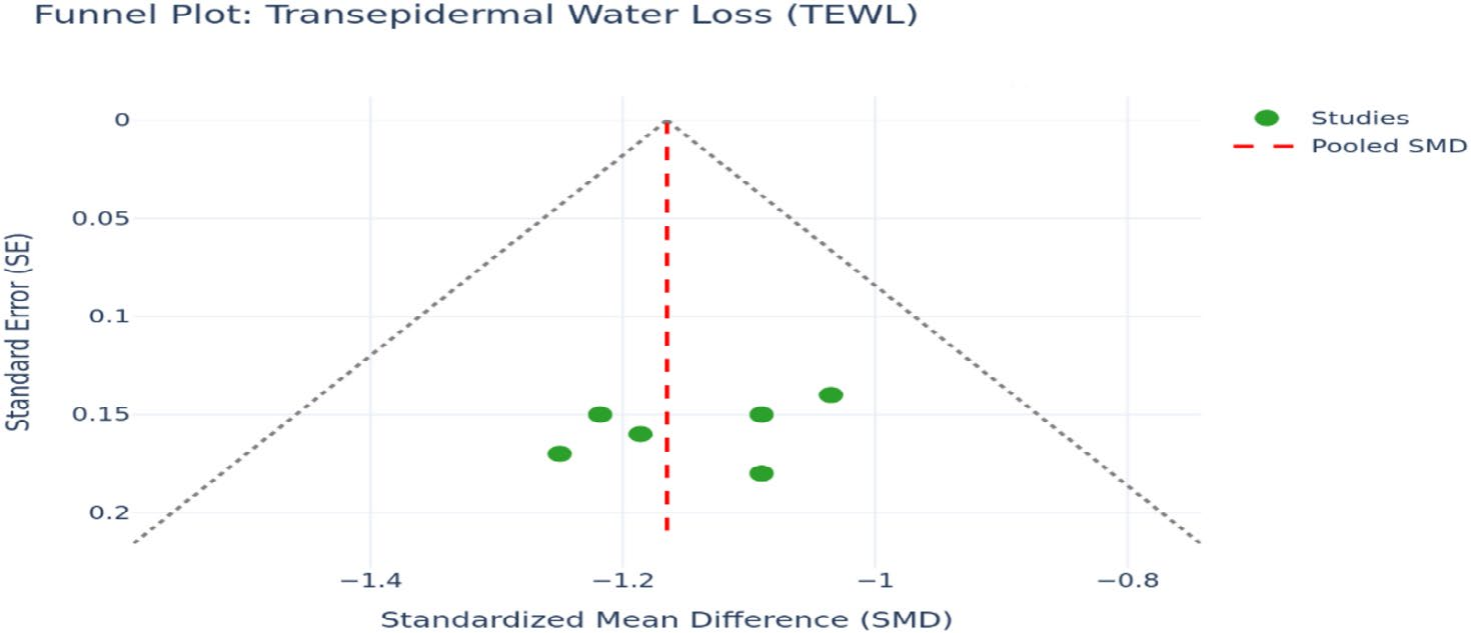
Funnel plot for TEWL outcome (*k* = 6). Visual inspection shows a relatively symmetric distribution of study effect sizes around the pooled estimates. Vertical red line = pooled SMD; dashed gray lines = 95% pseudo‐confidence interval limits.

Visual assessment of publication bias was conducted using funnel plots for outcomes with *k* ≥ 5 studies. For skin hydration (*k* = 5), funnel plot inspection revealed asymmetry, with smaller studies reporting larger effect sizes (Figure 8), consistent with potential small‐study effects or publication bias. However, formal statistical tests for funnel plot asymmetry (e.g., Egger's regression test) require at least 10 studies per outcome according to Cochrane guidelines and were therefore not applicable. For TEWL (*k* = 6), the funnel plot showed a more symmetric distribution, though the sample size remained insufficient for formal testing (Figure 9).

The predominance of industry‐funded studies (87%) raises concerns about selective reporting of favorable outcomes. The absence of trial registrations or published protocols for some studies (*n* = 6) further limits assessment of selective outcome reporting bias.

### 3.7 GRADE Certainty of Evidence

The certainty of evidence for each outcome was assessed using GRADE methodology (Table 3). Key factors influencing ratings included:

- **Risk of bias:** Not serious overall (93% of studies rated low risk).
- **Inconsistency:** Serious for hydration, elasticity, and wrinkles (*I* ^2^ > 75%); not serious for TEWL (*I* ^2^ < 10%).
- **Indirectness:** Not serious (populations, interventions, and outcomes aligned with review question).
- **Imprecision:** Serious for elasticity (*k* = 2, wide CI crossing null); not serious for other outcomes.
- **Publication bias:** Suspected for hydration (asymmetric funnel plot); not assessable for others (*k* < 10).

**TABLE 3:** GRADE summary of findings: Certainty of evidence.

Outcome	Studies (N)	SMD [95% CI]	*I* ^2^	*p*	Certainty
Skin Hydration	5 (402)	1.208 [0.410, 2.006]	87.5%	0.003	⊕ ⊕ ◯◯ Low^a^, ^b^
TEWL	6 (453)	−1.165 [−1.402, −0.927]	4.7%	< 0.0001	⊕ ⊕ ⊕◯ Moderate^c^
Skin Elasticity	2 (141)	0.930 [−0.179, 2.039]	82.9%	0.100	⊕◯◯◯ Very Low^a^, ^d^
Wrinkle Reduction	4 (287)	−1.299 [−1.736, −0.863]	80.0%	< 0.0001	⊕ ⊕ ◯◯ Low^a^

*Note:* GRADE summary of findings. SMD = standardized mean difference (Hedges' g). ⊕ ⊕ ⊕ ⊕ = High certainty; ⊕ ⊕ ⊕◯ = Moderate; ⊕ ⊕ ◯◯ = Low; ⊕◯◯◯ = Very low.

^a^ Downgraded for serious inconsistency (high *I* ^2^).

^b^ Downgraded for suspected publication bias.

^c^ Downgraded for some indirectness (variety of probiotic strains and intervention duration).

^d^ Downgraded for serious imprecision (*k* = 2, wide CI) and inconsistency.

**Summary:** TEWL had the highest certainty (moderate), reflecting consistent effects with low heterogeneity. Skin hydration and wrinkle reduction were rated low certainty due to high heterogeneity and suspected publication bias. Skin elasticity was rated very low certainty due to insufficient data (*k* = 2) and wide confidence intervals.

## 4 Discussion

### 4.1 Summary of Main Findings

This systematic review and meta‐analysis of 15 RCTs (867 healthy adults) provides the most comprehensive evidence to date on the effects of oral probiotic supplementation on skin outcomes. Our principal findings are as follows:

1. **Oral probiotics significantly improve skin hydration** (SMD = 1.208, *p* = 0.003), with a large effect size but high heterogeneity (*I* ^2^ = 87.5%). Certainty of evidence: Low.
2. **Oral probiotics significantly reduce TEWL** (SMD = −1.165, *p* < 0.0001), indicating enhanced skin barrier function. This effect was highly consistent across studies (*I* ^2^ = 4.7%). Certainty of evidence: Moderate.
3. **Oral probiotics significantly reduce wrinkle severity** (SMD = −1.299, *p* < 0.0001), though heterogeneity was high (*I* ^2^ = 80.0%). Certainty of evidence: Low.
4. **Evidence for improved skin elasticity is inconclusive** (SMD = 0.930, *p* = 0.100), with only two studies and high heterogeneity. Certainty of evidence: Very low.
5. **Oral probiotics are safe and well tolerated**, with no serious adverse events reported and mild gastrointestinal symptoms occurring at similar rates in probiotic and placebo groups.

Subgroup analysis suggested that interventions lasting ≥ **8 weeks** may confer greater benefits for skin hydration, though this finding requires confirmation in larger datasets. Strain‐specific effects could not be reliably assessed due to limited inter‐strain variability in the included studies.

### 4.2 Interpretation in Context of Existing Literature

Our findings align with emerging evidence on the gut‐skin axis and probiotic mechanisms. Preclinical studies have demonstrated that oral probiotics modulate systemic immune responses, reduce circulating inflammatory cytokines (e.g., IL‐6, TNF‐α), and enhance production of short‐chain fatty acids (SCFAs) such as butyrate, which indirectly influence skin barrier function and hydration [29, 30]. Specific strains like *Lactobacillus plantarum* and *Lactococcus lactis* have been shown to upregulate ceramide synthesis and filaggrin expression in keratinocytes, critical for maintaining stratum corneum integrity [31].

A previous narrative review by Gueniche et al. [8] suggested potential benefits of probiotics for skin health but lacked quantitative synthesis [17]. A 2020 systematic review by Kober and Bowe focused primarily on acne and atopic dermatitis, not healthy adult populations or cosmetic outcomes like hydration and wrinkles [10]. Our work fills this gap by providing pooled effect estimates with GRADE certainty ratings specifically for cosmetic and barrier function outcomes in non‐diseased populations.

The high heterogeneity observed for hydration and wrinkles is expected given the diversity of probiotic strains, dosages, and measurement techniques. Despite these variations, we deemed quantitative pooling appropriate because all interventions rely on the same fundamental mechanism—gut microbiome modulation to influence the gut‐skin axis—and evaluated identical physiological constructs. Consequently, the pooled estimates should be viewed as an “average” class effect of oral probiotics on skin parameters, rather than a precise expected magnitude of benefit for any specific commercial strain or regimen.

### 4.3 Mechanisms of Action

The biological plausibility of oral probiotics improving skin outcomes rests on several proposed mechanisms:

1. **Immunomodulation:** Probiotics enhance gut epithelial barrier integrity, reducing systemic lipopolysaccharide (LPS) translocation and attenuating low‐grade inflammation. This “leaky gut” hypothesis links gut dysbiosis to systemic inflammation, which manifests as skin barrier dysfunction and accelerated aging [32].
2. **Antioxidant effects:** Certain probiotic strains produce antioxidant enzymes (superoxide dismutase, catalase) that scavenge reactive oxygen species (ROS), mitigating oxidative stress‐induced collagen degradation and lipid peroxidation in the skin [33].
3. **Ceramide and lipid modulation:** Probiotics stimulate endogenous ceramide synthesis via regulation of sphingolipid metabolism, enhancing stratum corneum lipid lamellae structure and reducing TEWL [34].
4. **Neuroendocrine signaling:** The gut‐brain‐skin axis involves probiotic modulation of the hypothalamic–pituitary–adrenal (HPA) axis, reducing cortisol‐mediated skin barrier disruption and inflammation [35].
5. **Direct microbial metabolites:** Probiotics produce SCFAs (butyrate, propionate, acetate), which have anti‐inflammatory properties and may indirectly influence skin via systemic circulation [36].

These mechanisms are not mutually exclusive and likely operate synergistically. Strain‐specific effects remain poorly understood and merit targeted mechanistic studies.

### 4.4 Limitations

Several limitations warrant consideration:

1. **High heterogeneity:** Skin hydration (*I* ^2^ = 87.5%) and wrinkle (*I* ^2^ = 80.0%) outcomes exhibited substantial variability. This likely reflects diversity in probiotic strains, doses, durations, and participant characteristics. Random‐effects models account for heterogeneity statistically, but clinical interpretation remains complex.
2. **Small number of studies per outcome:** Skin elasticity (*k* = 2) and wrinkles (*k* = 4) had limited data, reducing statistical power and precluding robust subgroup analyses. Funnel plot asymmetry for hydration (*k* = 5) suggests possible publication bias, but formal tests were not applicable (*k* < 10).
3. **Industry funding:** Thirteen of 15 studies (87%) were funded or supported by probiotic manufacturers. While most declared conflicts of interest may bias study design (e.g., favorable strain selection) and selective publication of positive results.
4. **Lack of strain‐specific data:** Subgroup analysis by probiotic genus (*Lactobacillus*, *Bifidobacterium*, etc.) was underpowered due to predominance of *Lactobacillus* strains. Optimal strains, doses, and formulations for specific skin outcomes remain unclear.
5. **Short intervention durations:** Most studies lasted 4–12 weeks. Long‐term durability of probiotic effects on skin is unknown. Chronic supplementation may be required to sustain benefits, but no studies assessed outcomes beyond 24 weeks.
6. **Measurement variability:** While all studies used validated instruments (Corneometer, Tewameter, Cutometer), subtle differences in device models, calibration protocols, anatomical measurement sites, and environmental conditions (temperature, humidity) may have introduced variability.
7. **Generalizability:** Participants were predominantly young to middle‐aged females (75%–100%) with dry or normal skin. Effects may differ in males, older adults, individuals with oily or sensitive skin, or those with dermatological conditions.
8. **Trial registration and protocol availability:** Only 9 of 15 studies (60%) reported trial registration (e.g., UMIN, NCT), limiting assessment of selective outcome reporting bias. Pre‐specified analysis plans were often unavailable.
9. **Clinical interpretability of effect sizes:** We utilized standardized mean differences (Hedges' g) to pool data across heterogeneous measurement scales. While the resulting effect sizes appear large, SMDs are unitless and can be difficult to interpret clinically. Statistical back‐translation into raw Corneometer or Tewameter units was not feasible, as the included trials utilized heterogeneous measurement scales and instruments that preclude standardization. Large pooled effect sizes in nutritional interventions derived from a small number of trials frequently overestimate true clinical benefits due to small‐study effects. Therefore, the magnitude of these effects should be interpreted cautiously.

### 4.5 Implications for Practice

Despite limitations, our findings have practical implications:

- **For clinicians and dermatologists:** Oral probiotics, particularly *Lactobacillus* and *Bifidobacterium* strains, may serve as adjunctive interventions for improving skin hydration and barrier function in healthy adults with dry skin or mild aging concerns. Probiotics are safe, with minimal adverse effects, making them suitable for long‐term use.
- **For cosmetic and nutraceutical industries:** Our results support the inclusion of evidence‐based probiotic strains (e.g., *Lactobacillus plantarum* , *Lactococcus lactis* ) in oral beauty supplements (nutricosmetics). Marketing claims may be best offered to emphasize barrier function (TEWL reduction) given the moderate certainty evidence.
- **For consumers:** Oral probiotics may complement topical skincare regimens for individuals seeking hydration and anti‐aging benefits. Expectations should be realistic; effects are modest and require consistent use over ≥ 8 weeks.

### 4.6 Implications for Research

Future research should address the following priorities:

1. **Large, well‐powered RCTs:** Adequately powered trials (*N* > 100 per arm) with pre‐registered protocols are needed to confirm findings, reduce heterogeneity, and enable strain‐specific comparisons.
2. **Strain‐specific studies:** Head‐to‐head comparisons of different probiotic genera and species (e.g., *Lactobacillus plantarum* vs. *Bifidobacterium longum* ) are required to identify optimal strains for specific skin outcomes.
3. **Dose–response trials:** Few studies systematically varied CFU doses. Dose‐ranging trials (e.g., 10^9^, 10^10^, 10^11^ CFU/day) would inform optimal dosing strategies.
4. **Long‐term follow‐up:** Studies extending beyond 12 weeks, with post‐intervention washout phases, would clarify durability of effects and need for continuous supplementation.
5. **Mechanistic studies:** Translational research integrating biomarker assessments (e.g., plasma cytokines, fecal SCFA, skin microbiome profiling, epidermal ceramide quantification) with clinical outcomes would elucidate mechanisms and predict responders.
6. **Subpopulation analyses:** Studies enrolling diverse populations (males, older adults, different skin types, ethnic/racial groups) are needed to assess generalizability.
7. **Standardized outcome measures:** Consensus on core outcome sets for skin hydration, barrier function, and aging (e.g., standardized Corneometer protocols, TEWL measurement conditions) would improve comparability across trials.
8. **Combination interventions:** Trials testing oral probiotics combined with topical prebiotics or postbiotics may identify synergistic effects.

## 5 Conclusion

This systematic review and meta‐analysis provides low‐to‐moderate certainty evidence that oral probiotic supplementation may be associated with improvements in skin hydration and transepidermal water loss (TEWL) in healthy adults. The most consistent and robust evidence supports reductions in TEWL, indicating enhanced skin barrier function. Effects on wrinkle reduction show a positive trend but require confirmation in larger, more homogeneous studies, while evidence for improved skin elasticity remains inconclusive. Oral probiotics are safe and well‐tolerated. Findings should be interpreted cautiously due to substantial heterogeneity, small outcome‐specific trial numbers, and frequent industry sponsorship. Future research should prioritize large, strain‐specific RCTs to establish definitive conclusions on optimal strains, doses, and clinical significance.

## Author Contributions

Amin Shehni Nezhadpour: Conceptualization, methodology, systematic search, interpretation of results, quality assessment, writing – original draft, project administration, overall supervision, and final approval. Emadeddin Hemadi: Data extraction, quality assessment, and writing – review and editing. MohammadPouya Roghanian: Data curation, interpretation of results, and writing. Saba Shahinzadeh: Data extraction, quality assessment, and writing. Mehdi Makvandi: Data extraction, quality assessment, and writing. All authors reviewed and approved the final version of the manuscript and agree to be accountable for all aspects of the work.

## Funding

The authors have nothing to report.

## Conflicts of Interest

The authors declare no conflicts of interest.

## Supporting information


**Figure S1:** Leave‐one‐out sensitivity analyses for all meta‐analyzed outcomes. (A) Skin Hydration (*k* = 5). (B) TEWL (*k* = 5). (C) Wrinkle Reduction (*k* = 4). Blue diamonds = pooled estimate when indicated study is excluded; red diamond = full analysis. All outcomes remained statistically significant in every iteration.

**Table S1:** jocd71124‐sup‐0002‐TableS1.csv. RoB: Overall, methodological quality was high.

## Data Availability

The data that support the findings of this study are available from the corresponding author upon reasonable request.
