# Supplementary material for: Oral Probiotics for Improving Skin Hydration, Elasticity, Transepidermal Water Loss, and Wrinkle Reduction in Adults: A Systematic Review and Meta‐Analysis of Randomized Controlled Trials
Source: J Cosmet Dermatol. 2026 Aug 14;25(8):e71124. doi: 10.1111/jocd.71124 (PMC13474133; doi:10.1111/jocd.71124)
Supplement: Supplementary file 1 [file JOCD-25-e71124-s002.docx]

| Study ID | Randomization Process | Deviations from Interventions | Missing Data | Outcome Measurement | Selection of Results | Overall RoB | Randomization Notes | Blinding Notes | Attrition Notes |
| --- | --- | --- | --- | --- | --- | --- | --- | --- | --- |
| Uehara_2024 | Low | Low | Low | Low | Low | Low | Adequate random sequence | Double-blind design | 9.1% attrition, acceptable |
| Lee_2022 | Low | Low | Low | Low | Low | Low | Randomized adequately | Double-blind maintained | 0% attrition |
| Kimoto_2014 | Low | Low | Low | Low | Low | Low | Weak Method description | Adequately blinded | No attrition reported |
| Gueniche_2014 | Low | Low | Low | Low | Low | Low | Stratified randomization | Double-blind, evaluator blind | 3.1% attrition, balanced |
| Saito_2017 | Low | Low | Low | Low | Low | Low | Randomization computer-generated | Double-blind maintained | 3.3% attrition |
| Nobile_2025 | Low | Low | Low | Low | Low | Low | Proper randomization | Double-blind protocol | 0% attrition |
| Huuskonen_2022 | Low | Low | Low | Low | Low | Low | Stratified randomization | Triple-blind protocol enforced | 1.3% attrition |
| Peng_2022 | Low | Low | Low | Low | Low | Low | Randomized | Assessor blind | No attrition |
| Miyazawa_2017 | Low | Low | Low | Low | Low | Low | Adequate randomization | Double-blind maintained | No attrition |
| Nagino_2018 | Low | Low | Low | Low | Low | Low | Randomized design | Double-blind protocol | No attrition |
| Lee_2015 | Low | Low | Low | Low | Low | Low | Randomized | Double-blind | No attrition |
| Alves_2021 | Low | Low | Low | Low | Low | Low | Randomized | Blind assessor | No attrition |
| Togawa_2023 | Low | Low | Low | Low | Low | Low | Randomization stratified | Double-blind protocol | No attrition |
| Rybak_2023 | Low | Low | Low | Low | Some concern | Some concern | Randomized | Assessor blinded, participants not | No attrition |
| Alves_2022 | Low | Low | Low | Low | Low | Low | Randomized crossover | Double-blind crossover | No attrition |

**Supplementary_Table_1_RoB :** Overall, methodological quality was high.

- **Randomization process:** All 15 studies (100%) were rated low risk, with adequate random sequence generation and allocation concealment. One study (Kimoto-Nira et al., 2014) has weak reporting of the randomization methods but seems adequately randomized.
- **Deviations from intended interventions:** All 15 studies (100%) were rated low risk. All were double-blind (participants, personnel, and outcome assessors), and per-protocol or intention-to-treat (ITT) analyses were appropriately conducted.
- **Missing outcome data:** All 15 studies (100%) were rated low risk. Attrition rates were low (range: 0%–9.1%), with no evidence of differential dropout or bias due to missing data.
- **Measurement of the outcome:** All 15 studies (100%) were rated low risk. Outcome assessors were blinded, and objective measurement instruments (Corneometer, Tewameter, Cutometer, profilometry) were used consistently.
- **Selection of reported results:** 14 studies (93%) were rated low risk, with pre-specified outcomes and analysis plans reported. One study (Rybak et al., 2023) raised "Some concerns" due to incomplete reporting of secondary outcomes and lack of trial registration documentation.
- **Overall risk of bias:** 13 studies (93%) were judged to have low overall risk of bias, and one study (Rybak et al., 2023) was rated "Some concerns

Supplementary Figure 1. Leave-one-out sensitivity analyses for all meta-analyzed outcomes.

(A) Skin Hydration (k=5). (B) TEWL (k=5). (C) Wrinkle Reduction (k=4). Blue diamonds = pooled estimate when indicated study is excluded; red diamond = full analysis. All outcomes remained statistically significant in every iteration.
